# Clinical Cases

**Published:** 1889-12

**Authors:** George Logan

**Affiliations:** Ottowa, Ont., Canada


### CLINICAL CASES.

George Logan, M. D., Ottowa, Ont., Canada.

Case I.—Boy, six years old. Called to see him on September 14th, 1888. Found him in bed, complaining of sore throat.

On examination, found diphtheritic membrane covering the entire convexity of the left tonsil, with considerable redness and swelling of the adjoining part of the soft palate, considerable fever, and some enlargement of the gland of the left side of the neck; breath offensive. It had the appearance of being a decidedly malignant case. He had been ill two days before I was called. Lachesis$^{200}$, five or six pellets in quarter cupful of water, was taken at one dose, five or six pellets of Sac. lac. in half cupful of water—two teaspoonfuls one hour apart while awake until my return. Fresh air and isolation. On my return visit I found the disease arrested, and the boy said he felt better. Continued Sac. lac.

On the third day the left tonsil was much better. Continued Sac. lac.

Fourth day.—Left tonsil nearly well, or free from membrane; right tonsil partly covered by membrane. Complains of soreness of the right side. Continued Sac. lac.

Fifth day.—Right tonsil covered by membrane, but less redness and swelling than the left side had presented at first. Continued Sac. lac.

Sixth day.—Right tonsil worse, membrane extending to adjoining soft palate, with increased redness and swelling; did not feel so well. Right gland swollen. Gave three or four pellets of Lachesis as before, one dose, and continued Sac. lac. two hours apart.

Seventh day.—Decided improvement; spread of membrane arrested, and disappeared in a day or so afterward, followed by recovery.

Case II.—September, 1887. Was called to see a boy aged three years; had been ill two or three days; found membrane on both tonsils, and the soft palate near the tonsils involved; dark redness and swelling of tonsils and palate; considerable fever and difficulty of swallowing; glands on both sides enlarged, and breath fœtid. From what I could learn, the disease began on the left side; all the symptoms pointed to Lachesis, which was given—five or six pellets in half a cupful of water—two teaspoonfuls each hour for three or four times, then three hours apart until my next visit.

Second visit.—No worse. Continued the doses three hours apart.

Third visit.—Decided improvement. Sac. lac. three hours apart. In two or three days he was all right again.

Case III.—Boy, four years old had been ill three or four days; found him in bed; both tonsils covered by diphtheritic membrane; dark red swelling of the tonsils, soft palate, and fauces; glands much swollen, and very fœtid breath; great difficulty in swallowing. From all appearances, a very malignant case. Could only infer from evidence obtained that the left tousil was first involved.

Lachesis$^{200}$, one hour apart for three times, then three hours.

Second visit.—No improvement that I could detect; could not find any other remedy that covered the case better than Lachesis, which was continued three hours apart.

Third day.—Much worse; larynx involved; dry, croupy cough; stupor; refused medicine and food. Kali bich.$^{2c}$ one hour apart for three times, then three hours apart. Died the following morning. The most prominent symptoms in the above case were purple hue of the palate and fauces, intense fœtor, difficulty of swallowing, sensitiveness to external pressure, cannot bear anything to touch the neck. Worse on waking from sleep.

This case was one of the most malignant that I have seen in my practice of over thirty years.

A few years ago I reported six fatal cases in all during twenty-six years.

I then expressed the opinion that there is a degree of malignity, so far as I know, which I consider incurable.

After four years of further experience, I venture the same opinion—that there is a degree of malignity, only at long intervals met with, which is beyond the powers of nature, assisted by medication, to overcome.

If there are any men here who have had a large experience in such cases and have never lost one, I will be glad to hear from them. Two or three cases of success or failure are not sufficient to base a rule upon. "One sparrow is no sure sign of spring." It requires a large number of cases to warrant our forming an opinion or experience which will be a guide to others.

I have selected these cases from perhaps thirty cases treated, four of which proved fatal.

I hope some member of this Institute will be able to show a better record.

CASE IV.—Female child, four years old, was brought to my office. She presented a most repulsive appearance. The case was one of chronic eczema, of three years' standing. When less than a year old the eruption appeared on the face and head in the usual form, going through all the stages of eczema, papulation, vesiculation, pustulation, incrustation, and in some parts of the skin, desquamation. The eruption covered the entire surface of the body from the head to the feet. It had become infilterated and thickened, divided by an innumerable number of fissures running in all directions over the surface of the body like so many rivulets made to carry off the exudation accumulating on the surface. The child had a vulgar, filthy look, was intensely irritable, day and night. From some of the fissures mentioned blood was running at the time I saw the child.

I refused to have anything to do with the case unless I was allowed several months to conduct the cure. With this understanding I gave Sulphur$^{30}$, a dose each morning for three times, then Sac. lac. for three weeks, at which time they were to bring the child again. At the end of this time the case looked worse, if that could be possible, than before.

Continued the placebo for three weeks more and then to report. At the end of this time there was an improvement, the skin was less inflamed, the child was less irritable, the mother said she had given her much less trouble then before.

Continued Sac. lac. for three weeks more, at the end of which time a decided improvement was visible. Continued placebo until the case seemed to remain stationary, when I gave one dose of Sulphur$^{200}$, followed by Sac. lac.

From this time the improvement went on uninterruptedly until the child was perfectly well—a handsome young girl. I may mention that, as usual in such cases, she went the rounds of three or four medical men, each having his pet mode of procedure—cod liver oil, astringents, *ad infinitum.*

